# Identifying populations at high risk of malaria: a mixed-methods case–control study to inform targeted interventions in Senegal

**DOI:** 10.1186/s12936-024-05219-z

**Published:** 2024-12-18

**Authors:** Tidiane Thiam, Demba Kande, Henry Ntuku, Caterina Guinovart, Natalie Galles, Laura Merriman, Moustapha Cissé, Abiboulaye Sall, Ndack Diop, Aichatou Barry Diouf, Mama Moussa Diaw, Mamadou Diop, Baba Camara, Niene Seck, Aliou Ndour, Yakou Dieye, Jennifer Smith, Adam Bennett

**Affiliations:** 1 PATH, Seattle, USA; 2 Senegal Ministry of Health, National Malaria Control Programme, Dakar, Senegal; 3 Malaria Elimination Initiative, University of California San Francisco, San Francisco, CA USA; 4 Barcelona Institute for Global Health (ISGlobal), Barcelona, Spain

**Keywords:** Malaria, Risk factors, High risk populations

## Abstract

**Background:**

Senegal has made significant progress in reducing the burden of malaria, but transmission remains highly heterogeneous, with specific population subgroups likely at higher risk. Consultations with the National Malaria Control Programme (NMCP) and a review of available data identified nomadic pastoralists, gold miners, and Koranic school students as potential high-risk populations (HRPs). This study aimed to evaluate whether these populations are at higher risk of malaria and better characterize their exposure patterns to inform the design of targeted intervention strategies.

**Methods:**

A mixed-methods study was conducted in the districts of Ranérou, Kaolack, and Saraya between November 2020 and December 2021. A formative assessment including key informant interviews (KII) and focus group discussions (FGD) was conducted with non-HRP and HRP members (nomadic pastoralists, gold miners, Koranic school students). A health facility-based case–control study was then conducted in nine health facilities across the three districts. 501 confirmed malaria cases and 1002 non-malaria controls were frequency matched by age and sex with a ratio of 1:2. A standardized questionnaire was administered to collect sociodemographic information, including occupation, use of malaria prevention measures, mosquito exposure, and travel history. Multivariable logistic regression was used to identify malaria risk factors.

**Results:**

KIIs and FGDs indicated that nomadic pastoralists, gold miners and Koranic school students have high exposure to mosquito bites through outdoor sleeping, spending time outside at night and sleeping in informal structures, with important gaps in the coverage of indoor residual spraying (IRS) and long-lasting insecticidal nets (LLINs) and limited access to health services. Compared to controls, cases had higher odds of being a nomadic pastoralist (odds ratio (OR) 4.67 95% CI 1.96–11.11) or gold miner (OR 1.92 95% CI 1.20–3.07). No evidence was found of an association with being a Koranic school student (OR 1.39 95% CI 0.80–2.39).

**Conclusions:**

Nomadic pastoralists and gold miners in the study areas are at higher risk of malaria. Targeted interventions are needed to cover gaps in malaria prevention coverage and access to health services.

**Supplementary Information:**

The online version contains supplementary material available at 10.1186/s12936-024-05219-z.

## Background

Senegal has made substantial and sustained progress in reducing malaria morbidity and mortality over the past decade thanks to the successful scale-up and effective implementation of malaria control interventions, including long-lasting insecticide-treated nets (LLINs) and improved case management. The national parasite prevalence in children under 5 years of age has dropped from 5.7% in 2008–09 to 0.4% in 2017 [1–4]. Similarly, malaria mortality in children under 5 years of age has decreased by 51% from 4 malaria deaths per 100,000 inhabitants in 2015 to 2 deaths per 100,000 inhabitants in 2019 [5]. Despite the low parasite prevalence at national level, there is still substantial spatial heterogeneity in malaria transmission at the subnational level with the annual parasite index (API) ranging from less than 5 cases per 1000 population per year in the northern regions to more than 30 cases per 1000 population per year in the southern regions [6].

The impact of the successful malaria control intervention in recent years in Senegal has likely also affected the distribution of malaria cases with specific population subgroups at higher risk of infection, as evidenced in malaria elimination settings globally [7–10]. Populations at higher risk can contribute to sustaining malaria transmission dynamics leading to high incidence in certain areas and present challenges for malaria control and elimination efforts. Reducing malaria burden and ultimately achieving malaria elimination will require the development of appropriate targeted interventions tailored to the needs of underserved groups with varying exposures, intervention gaps, opportunities and barriers to delivering interventions. Identifying specific high-risk populations and risk factors is necessary to adapt surveillance systems and targeted responses [11–13]. Consultations with the NMCP and a review of available data identified three population groups who potentially have higher malaria risk and lower access to malaria interventions and healthcare, including nomadic pastoralists, gold miners, and boarding students in Koranic schools. Few studies have shown high malaria burden in these specific groups and have identified associated risk factors including travel and low use of malaria prevention interventions [14–16]. A mixed-methods study was conducted to evaluate whether these populations are at higher risk of malaria and to better understand their exposure patterns, with the aim of informing the design of targeted intervention strategies.

## Methods

### Study setting and population

**Fig. 1:**
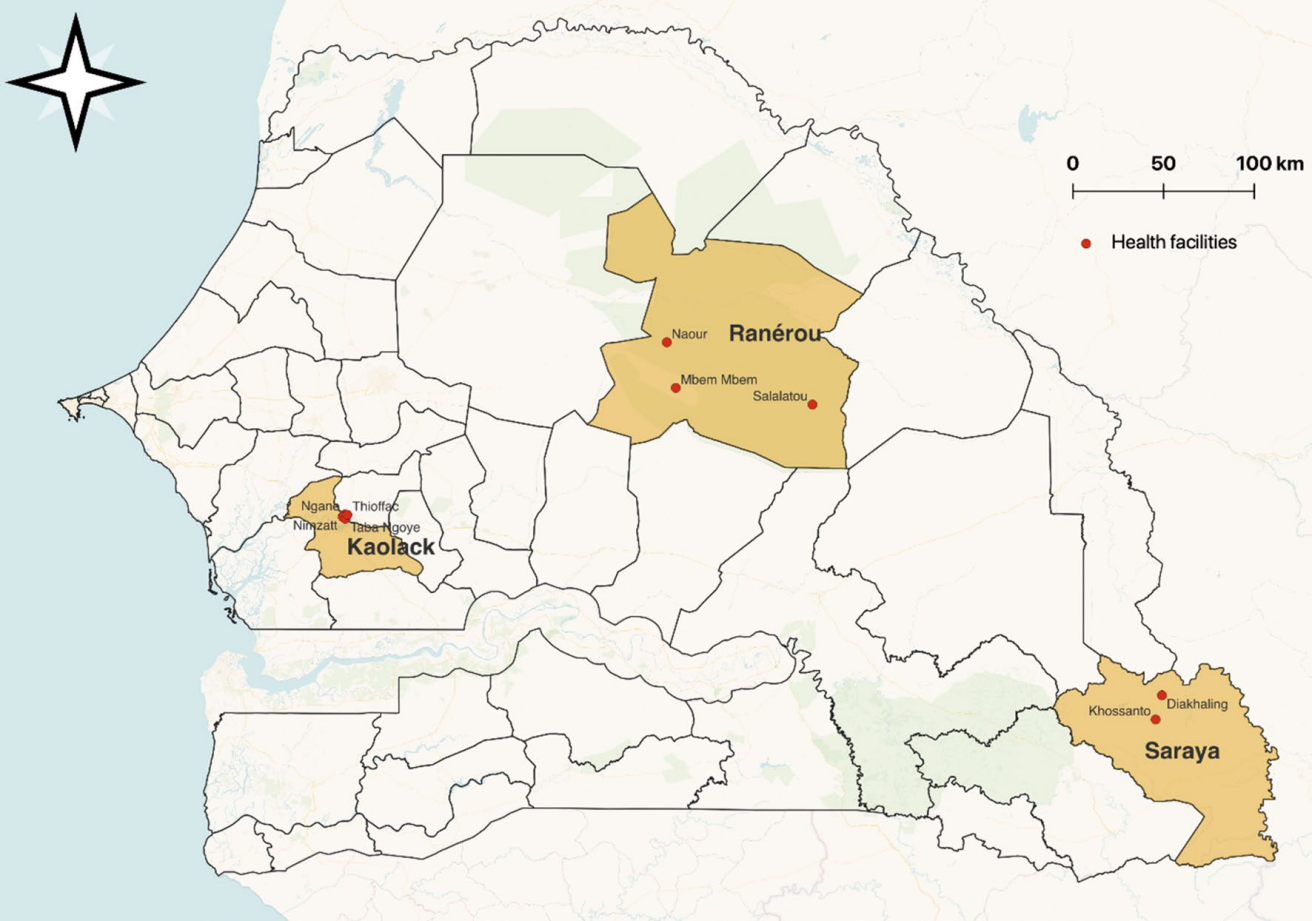
Map of Senegal with health facilities included in the study

The study was conducted in 2020 and 2021 in the health districts of Ranérou, Kaolack, and Saraya in Senegal, which were selected based on the presence of the three potential high-risk groups (Fig. 1). With a population of 60,000 inhabitants, the health district of Ranérou is located in the northeast and is the primary resident district of nomadic pastoralists who travel with their livestock seasonally (Nov–June) to the southern regions in search of greener pastures, where malaria transmission is higher. They return to Ranérou during the rainy season (July–Oct). Ranérou had a reported malaria incidence of 35 per 1000 population in 2019. Kaolack health district is in the centre of the country with a population of 355,000 inhabitants. It has the largest number of traditional Koranic schools, called "Daaras", and boarding learners, called "Talibes", in Senegal. Kaolack had a reported malaria incidence of 7.7 per 1000 population in 2019. Saraya district is in the southeast with a population of 58,000 inhabitants. Artisanal gold mining sites in the area attract gold miners from Senegal and neighbouring countries. In 2019, the incidence of malaria in Saraya was 379.8 per 1000 population [6]. In all three districts, malaria transmission is almost entirely due to *Plasmodium falciparum* and highly seasonal, with the typical high season running from August to January during and just after the rainy season.

### Study design

A mixed-methods study combining a qualitative formative assessment with key informant interviews (KII) and focus group discussions (FGD), and a health facility-based prospective case–control study was conducted.

### Study recruitment

#### Formative assessment

The formative assessment was conducted in November 2020, shortly before the start of the case–control study, in villages in the catchment areas of the study health facilities based on known presence of target populations after consultation with the community. KIIs were conducted with members of the target populations (nomadic pastoralists, gold miners and Koranic school students), and with community members particularly knowledgeable about these groups, whereas FGDs were conducted with members of the target populations. Participants were eligible for KII and FGD if they were over 18 years of age, spoke French or Wolof or another language spoken by study staff, and provided consent. Purposive and referral sampling were used to recruit participants. Participants were recruited from villages, Daaras, gold mines, and health facilities, or community members who provide services to or are knowledgeable about the target populations (e.g., health facility staff, community health workers, community leaders, employers of gold miners, Daara chiefs, parents of Koranic school students, well managers, artisanal gold mine owners). A total of 33 KIIs, including 24 with members of the target populations, and seven FGDs, were conducted in the three districts and for the three population groups. The total number of KIIs and FGDs conducted by population group is shown in Table 1.

**Table 1:** Number of key informant interviews and focus group discussions recruited by district

	Saraya	Ranérou	Kaolack	Total
Key informant interviews (N)	15	6	12	33
Target HRPs’ members (n)	12	4	8	24
Community members (n)	3	2	4	9
Focus group discussions (N)	3	2	2	7

#### Case–control study

The prospective case–control study was conducted in nine health facilities (Fig. 1), three in Ranérou, four in Kaolack, and two in Saraya district. The selection of recruiting health facilities was informed by the qualitative assessment results based on the known presence of gold mining sites, Daaras and nomadic pastoralists in the catchment area. Participants were recruited from November 2020 to January 2021 in Kaolack and Saraya and from November 2020 to December 2021 in Ranérou. Cases were defined as patients of any age with uncomplicated malaria confirmed by a positive rapid diagnostic test (RDT) or blood smear at any of the selected health facilities during the study period, while controls were febrile patients with a negative malaria RDT or blood smear at the selected study health facilities. Exclusion criteria for cases and controls were severe illness and having spent less than 2 weeks in the district in the last 30 days. Controls were also excluded if they had a malaria diagnosis in the preceding month or had taken anti-malarials in the previous 14 days. Cases and controls were frequency matched at the facility level by age group and sex, with a ratio of 1 case to 2 controls. The study aimed to enroll a total of 500 cases and 1000 controls to detect an odds ratio of 2 for being a member of a high-risk group, based on the assumption of a 15% prevalence of high-risk occupation in the controls, with 80% power and a two-sided significance level of 5%.

### Data collection and management

#### Formative assessment

Semi-structured interview guides for KIIs and FGDs guides were developed to collect information on the different population groups, their movements during the year, travel patterns, treatment-seeking behaviors, barriers to accessing health services, and malaria prevention use and preferences. These interviews also gathered information for the implementation of malaria surveillance and control interventions targeting at-risk populations, including where and when to reach these populations and potential barriers to access. One staff member led the group discussions or individual interviews, and another took notes and created an audio recording. Both notes and audio recording were used to produce the interview summaries.

#### Case–control

Patients with non-severe disease confirmed positive or negative by RDT or microscopy for malaria were informed of the study by the health care provider and referred to a study staff based at the health facility to be screened for eligibility. Eligible individuals were invited to participate and, if written informed consent was obtained, information on RDT or microscopy results and malaria treatment was extracted from the patient's record. For all participants recruited as cases, the health care provider collected an additional blood sample by finger prick to prepare dried blood spots (DBS). Using the qualitative assessment results, a standard individual questionnaire was designed to collect demographic characteristics, occupation, individual and household malaria prevention measures (Indoor residual spraying (IRS), LLINs, Seasonal Malaria Chemoprevention (SMC)), treatment-seeking for previous fever episodes, travel history in the past four weeks, bed time and wake up time, and frequency and time spent outside at night. The presence of material goods in the household, such as radios, electricity, and various types of livestock was recorded. From this, a composite wealth index was created using principal components analysis (PCA) to determine socioeconomic status [17].

Android Tablets were used to fill out the electronic questionnaire, which was programmed using Open Data Kit (ODK, University of Washington & Google Foundation). Data were sent to a secure cloud-based server using a local cell phone network connection. Data were retrieved from the server for cleaning and analysis using Stata 16.0 (StataCorp, College Station, TX) following a pre-defined analysis plan.

### Data analysis

#### Formative assessment

For the formative evaluation, a rapid analytical approach, consistent with rapid assessment procedures [18], was used to quickly produce results that could inform the case–control study. Audio recordings were not transcribed. Iterative data analysis took place during and after the fieldwork; at the end of each day, the research team met to debrief and compare observations and interpretations of the data collected that day. The notes taken during debriefings helped to identify themes arising from the data and were used to produce summary reports. Data triangulation from observations, interview notes and recordings were used to ensure reliability and validity. Results are presented under three themes—(1) population description, (2) location, travel, and mobility, (3) malaria risk, prevention and access to health services.

#### Case–control

Potential risk factors for symptomatic confirmed malaria were assessed using logistic regression models with RDT result (positive/negative) as the dependent variable. Dummy variables were created for each of the three high-risk occupations of interest (pastoralists, Koranic school students and gold miners). Bivariate analysis was first conducted, and a multivariable model was constructed with all significant variables from the bivariate analysis (significance level of 0.05) and a priori risk factors. Group matching variables (age group, gender, and health facility) were included as covariates in all models. Other variables were added or removed stepwise while assessing for collinearity, and covariates that improved the quasi-likelihood of the model were retained. Key risk factors considered to be on the causal pathway for the association between high-risk occupation and clinical malaria (including ownership/use of LLIN, sleeping outdoors, spending time outdoors at night, having spent at least one night away from home, IRS and SMC) were considered mediators and not confounders and were not adjusted for in the main model. Models adjusting for these variables are presented in supplementary materials. Interactions were also assessed and the Akaike information criterion (AIC) was used to select the final model. The primary final model combined data from the three districts using a mixed-effects model with a fixed effect at the district level and random effects at the health facility level to account for clustering. District-specific models were also run, including only a fixed effect at the health facility level and are presented in supplementary materials. Results are presented as odds ratios with 95% confidence intervals and a significance level of 0.05. c

### Ethical considerations

The study protocol was approved by the University of California, San Francisco Human Research Committee (CHR), the National Health Research Ethics Committee of Senegal, and the PATH Research Ethics Committee. Participation in all research activities was voluntary. Written consent was obtained from all participants. For participants under 18 years of age, informed consent was obtained from a parent or guardian. Assent was obtained for adolescents aged 12 to 17 years, in addition to consent from a parent or guardian.

## Results

### Formative assessment

#### Description of the population

Nomadic pastoralists were mostly Senegalese men and women from Ranérou or other regions of Senegal, but also from other countries such as Mali and Mauritania. The nomadic pastoralists interviewed for the study were between 35–55 years of age. Gold miners who participated in this study were men and women between 20 and 35 years of age and mostly Senegalese, but also from other countries such as Mali, Guinea, and Burkina Faso. Koranic school students were mostly boys between 5 and 17 years of age, boarding in Daaras, under the responsibility of the school's master, called “Serigne Daara” or “Marabout”. The Koranic school students have other activities in the afternoon and evening such as small trade, searching for wood, begging for lunch and dinner, and participating in agricultural activities.

#### Location, travel, and mobility

Most nomadic pastoralists interviewed had their permanent residence in Ranérou and travelled south each year to the southern regions in search of pasture and water for their cattle, with the primary destination being the Tambacounda region. They generally leave their homes around November/December and return around July, travelling for approximately 15–30 days. Their routes usually follow the water points and local markets. The majority of nomadic pastoralists travelled by cart pulled by their cattle with their families.

Local gold miners live in the villages around the mines, whereas gold miners from other regions or neighbouring countries sleep in informal structures (“Niaffa”) made of reeds outside the villages. Senegalese gold miners return to their regions during the rainy season for agricultural activities, and during major religious holidays. Gold miners from other countries may return to their country several times during the year depending on earnings. Gold miners also reported moving to newly discovered gold mines. Koranic school students reported travelling back home during religious ceremonies and events, and family ceremonies such as baptisms or mourning. Some also reported moving during the rainy season to help with agricultural activities at the Marabout’s fields and return to the Daara after the harvest.

#### Malaria risk, prevention, and access to health services

All three population groups reported a high level of indoor and outdoor exposure to mosquitoes, with obvious gaps in intervention coverage. Nomadic pastoralists, especially young people, typically sleep outdoors to look after their cattle without using preventive measures. They have difficulty accessing LLINs due to their mobility and, because they sleep in makeshift structures also have difficulty hanging them. Gold miners work from 7AM to 6PM but reported spending a lot of time outside socializing in the evenings, especially in hot weather. Gold miners reported having low coverage of malaria prevention interventions, as they have low access to LLINs and Niaffas are not sprayable. Participants reported that breeding sites are common around the Niaffas, especially during the rainy season. Koranic school students spend time outside begging at night and sometimes sleep outside due to lack of space in the Daaras. Daaras are often unfinished structures, with open doors and windows. They usually share a sleeping space with 5–10 students per room mostly without using LLINs due to the nets being too small to cover large sleeping spaces, not having enough nets for everyone, or not being able to hang them properly. Eligible Koranic school students are normally targeted during SMC campaigns.

These three population groups have limited access to health services with group-specific barriers. Nomadic pastoralists indicated that the cost of prescriptions, social distance, and the opening hours of health posts are the main obstacles to accessing health services. Self-medication is very common. However, pregnant women and children are able to visit the health posts in stopover villages. Similarly, lack of time is the main obstacle to accessing health services for gold miners. They visit the health post on infrequent off-days, or in case of severe symptoms, and indicated a preference for traditional medicines. For Koranic school students, the Marabout makes the decision to seek treatment. Stigmatization, distance to health posts, and financial resources are the main reported obstacles to accessing health services.

### Case–control

#### Descriptive analysis

**Fig. 2:**
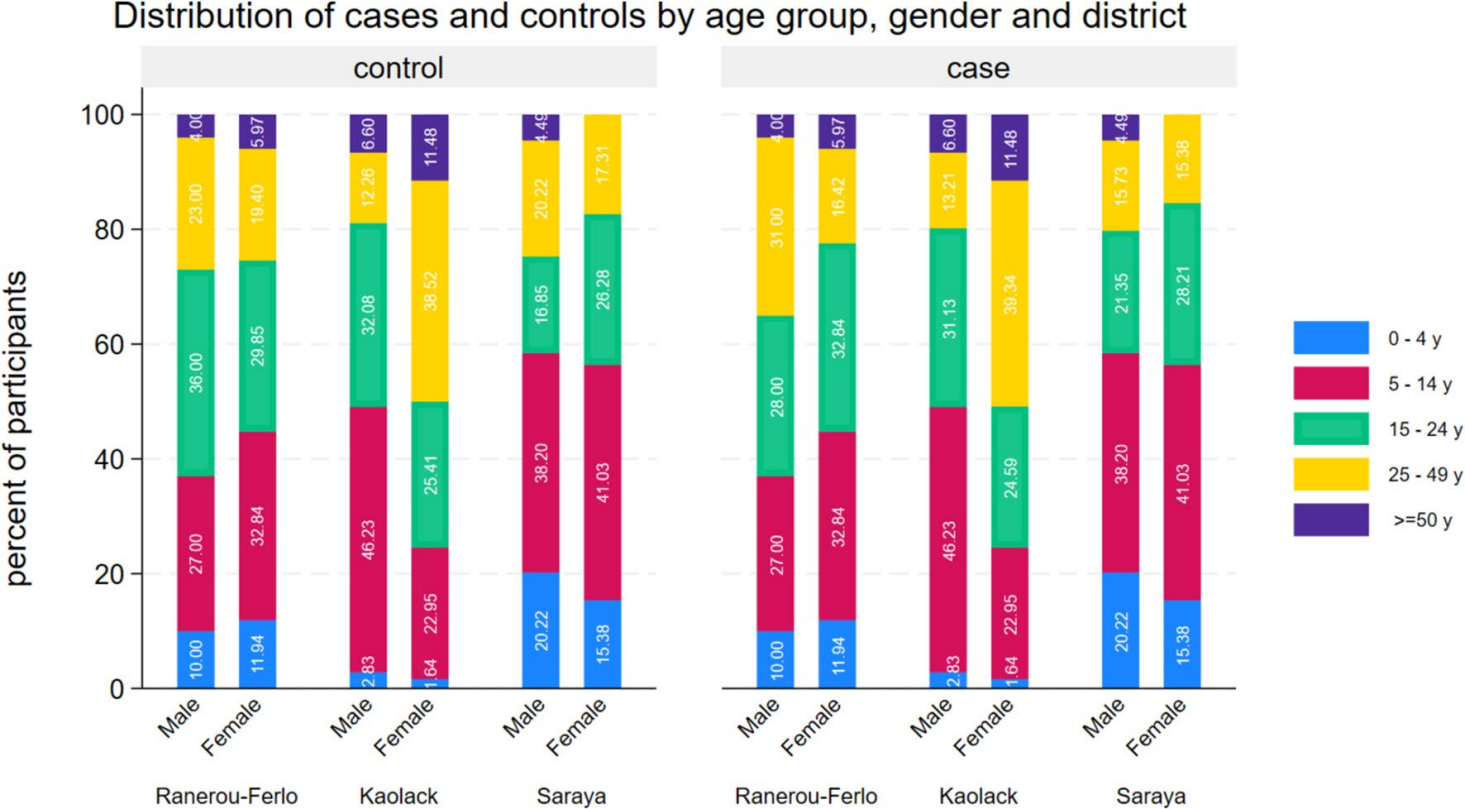
Distribution of cases and controls by age group, gender and district

A total of 501 cases and 1002 controls were recruited during the study. Distribution of study participants by gender, age group and district are shown in Fig. 2 and by sociodemographic characteristics in Table 3. Cases and controls were more likely to be male (59%) and the median age was 16 years (IQR 8–25 years for cases and 9–25 years for controls).

The characterization of malaria risk factors among high-risk groups vs non-high-risk group participants is shown in Table 2. Compared to non-HRP participants (54.0%), nomadic pastoralists (76.3%), gold miners (78.5%) and Koranic school students (86.3%) were more likely to be male. Nomadic pastoralists and gold miners had a lower education level, with 79.0% and 70.0%, respectively, reporting no formal education, compared to 45.7% in non-HRP participants The majority of nomadic pastoralists lived in informal structures (79.0% versus 14.1% in the non-HRP participants). Nearly half of nomadic pastoralists (44.7%) traveled in the last month whereas only 3.9% of Koranic school students, 2.3% of gold miners and 2.6% of the non-HRP participants did. The majority of nomadic pastoralists reported sleeping outside in the past month (79.0%) versus 14.7% of Koranic school students, 23.8% of gold miners and 18.5% of the non-HRP participants. LLIN ownership was similar between Koranic school students (77.5%), gold miners (78.5%) and the non-HRP participants (70.7%), but lower among nomadic pastoralists (39.5%). The same trend was observed with the LLIN use with 70.6% use among Koranic school students, 60.0% among gold miners, 56.0% in the non-HRP participants and 21.1% use among nomadic pastoralists. Only 50% of Koranic school learners and 58.2% of gold miners sought treatment for a fever episode compared to 91.6% of nomadic pastoralists and 75.3% of non-HRP participants.

**Table 2:** Characterization of malaria risk factors among HRPs compared to non-HRP participants

	Nomadic pastoralists (N = 38)	Koranic school students (N = 102)	Gold miners (N = 130)	Non-HRP participants (N = 1233)
	n (%)	n (%)	n (%)	n (%)
Gender				
Male	29 (76.3)	88 (86.3)	102 (78.5)	666 (54.0)
Female	9 (23.7)	14 (13.7)	28 (21.5)	567 (46.0)
Age group				
0–4 y	0 (0.0)	0 (0.0)	0 (0.0)	156 (12.7)
5–14 y	7 (18.4)	75 (73.5)	15 (11.5)	437 (35.4)
15–24 y	13 (34.2)	25 (24.5)	60 (46.2)	323 (26.2)
25–49 y	17 (44.8)	2 (2.0)	48 (36.9)	247 (20.0)
50 y and more	1 (2.6)	0 (0.0)	7 (5.4)	70 (5.7)
Education				
No formal education	30 (79.0)	45 (44.1)	91 (70.0)	563 (45.7)
Primary	7 (18.4)	22 (21.6)	28 (21.5)	312 (25.3)
Secondary	0 (0.0)	7 (6.9)	6 (4.6)	134 (10.9)
University	0 (0.0)	0 (0.00)	0 (0.0)	11 (0.9)
Koranic school	1 (2.6)	20 (19.6)	1 (0.8)	26 (2.1)
Pre-school	0 (0.0)	8 (7.8)	4 (3.1)	187 (15.2)
Residence structure				
Traditional permanent	7 (18.4)	1 (1.0)	61 (46.9)	287 (23.3)
Modern	1 (2.6)	97 (95.1)	64 (49.2)	772 (62.6)
Informal	30 (79.0)	4 (3.9)	5 (3.9)	174 (14.1)
Fever in the last 6 months				
Yes	12 (31.6)	2 (2.0)	55 (40.8)	252 (20.4)
No	26 (68.4)	99 (97.0)	53 (42.3)	867 (70.3)
Don’t know	0 (0.0)	1 (1.0)	22 (16.9)	114 (9.2)
Sought advice or treatment during last fever episode				
No	1 (8.3)	1 (50.0)	22 (40.0)	29 (11.5)
Yes, from formal health sector (health post, centre, hospital)	10 (83.3)	1 (50.0)	30 (54.6)	195 (77.4)
Yes, from community services (community health worker, case de santé, mobile outreach team)	1 (8.3)	0 (0.0)	2 (3.6)	20 (7.9)
Don’t know/remember	0 (0.0)	0 (0.0)	1 (1.8)	6 (2.4)
Pharmacy	0 (0.0)	0 (0.0)	0 (0.0)	2 (0.8)
Traveled in the last month				
No	21 (55.2)	98 (96.1)	127 (97.7)	1201 (97.4)
Yes	17 (44.8)	4 (3.9)	3 (2.3)	32 (2.6)
Slept outside in the last month				
No	8 (21.0)	87 (85.3)	99 (76.2)	1005 (81.5)
Yes	30 (79.0)	15 (14.7)	31 (23.8)	228 (18.5)
Weekly hours spent outside between 18 and 6 h in the past month				
No time spent outside	4 (10.5)	1 (1.0)	4 (3.1)	270 (21.9)
1–12 h	9 (23.7)	28 (27.5)	95 (73.1)	583 (47.3)
13–16 h	2 (5.3)	36 (35.3)	31 (23.8)	207 (16.8)
> 16 h	23 (60.5)	37 (36.2)	0 (0.0)	173 (14.0)
Structure sprayed last year (IRS)				
No	33 (86.8)	0 (0.0)	0 (0.0)	28 (2.3)
Yes	5 (13.2)	0 (0.0)	10 (7.7)	502 (40.7)
Not eligible	0 (0.0)	102 (100.0)	120 (92.3)	703 (57.0)
Own at least 1 LLIN				
No	23 (60.5)	23 (22.5)	28 (21.5)	361 (29.3)
Yes	15 (39.5)	79 (77.5)	102 (78.5)	872 (70.7)
Slept under LLIN last night				
No	30 (78.9)	30 (29.4)	52 (40.0)	542 (44.0)
Yes	8 (21.1)	72 (70.6)	78 (60.0)	691 (56.0)
Ratio LLIN per person				
Less than 1 LLIN for 2 people	32 (84.2)	73 (71.6)	64 (49.2)	871 (70.6)
At least 1 LLIN for 2 people	6 (15.8)	29 (28.4)	66 (50.8)	362 (29.4)
Number of people sharing sleeping space				
0–1	9 (23.7)	3 (2.9)	32 (24.6)	124 (10.1)
2–3	27 (71.0)	72 (70.6)	97 (74.6)	1057 (85.7)
> = 4	2 (5.3)	27 (26.5)	1 (0.8)	52 (4.2)
Seasonal malaria chemoprevention (SMC)				
Yes	0 (0.0)	27 (26.5)	3 (2.3)	139 (11.3)
No	0 (0.0)	20 (19.6)	5 (3.9)	141 (11.4)
Not applicable	38 (100.0)	55 (53.9)	122 (93.9)	953 (77.3)

#### Malaria risk factors

Table 3 shows the results of the bivariate and multivariable analyses with data from the three districts combined. In the multivariable analysis, the odds of being a nomadic pastoralist (OR 4.67 95% CI 1.96–11.11) or gold miner (OR 1.92 95% CI 1.20–3.07) was higher in cases than in controls. No evidence of association was found with being a Koranic school student (talibé) (OR 1.39 95% CI 0.80–2.39). Other significant risk factors for malaria infection included sharing the sleeping space with four people or more (OR 2.19 95% CI 1.18–4.05), having the main place of residence outside the study district (compared to main residence in the study district) (OR 3.27 95% CI 1.38–7.73) and being recruited during the high transmission season (OR 6.14 95% CI 3.67–10.26).

**Table 3:** Malaria risk factors—bivariate* and multivariable logistic regression model results (three districts combined)

	Cases n (%) (N = 501)	Controls n (%) (N = 1002)	OR* (95% CI)	*p*-value	Adjusted OR (95% CI)	*p*-value
Age group						
0–4 y	52 (10.38)	104 (10.38)	Ref.		Ref.	
5–14 y	178 (35.53)	356 (35.53)	1.07 (0.77–1.57)	0.725	0.83 (0.45–1.53)	0.546
15–24 y	139 (27.74)	282 (28.14)	0.99 (0.66–1.48)	0.978	0.78 (0.39–1.56)	0.479
25–49 y	106 (21.16)	208 (20.76)	0.96 (0.63–1.46)	0.874	0.68 (0.33–1.38)	0.286
≥ 50 y	26 (5.19)	52 (5.19)	0.91 (0.49–1.65)	0.758	0.71 (0.31–1.65)	0.427
Gender						
Male	295 (58.88)	590 (58.88)	Ref.		Ref.	
Female	206 (41.12)	412 (41.12)	0.92 (0.74–1.15)	0.508	1.07 (0.85–1.36)	0.574
Occupation—Nomadic pastoralist**						
Non-Nomadic pastoralist	473 (94.1)	992 (99.0)	Ref.		Ref.	
Nomadic pastoralist	28 (5.9)	10 (1.0)	8.27 (3.60–18.99)	0.000	4.67 (1.96–11.11)	0.000
Occupation—Gold miners**						
Non-gold miner	448 (89.42)	925 (92.32)	Ref.		Ref.	
Gold miner	53 (10.58)	77 (7.68)	1.65 (0.97–2.81)	0.061	1.92 (1.20–3.07)	0.007
Occupation—Koranic school student						
Non-Koranic school student	454 (90.62)	947 (94.5)	Ref.		Ref.	
Koranic school student	47 (9.38)	55 (5.49)	1.54 (0.83–2.88)	0.169	1.39 (0.80–2.39)	0.243
Education level						
No formal education	252 (50.30)	477 (47.60)	Ref.		Ref.	
Primary school	125 (24.35)	244 (24.35)	0.97 (0.70–1.34)	0.872	1.00 (0.71–1.41)	0.999
Secondary school	38 (7.58)	109 (10.88)	0.62 (0.39–0.96)	0.036	0.70 (0.44–1.11)	0.133
University	2 (0.40)	9 (0.90)	0 .41 (0.08–2.05)	0.280	0.40 (0.08–2.06)	0.272
Koranic school	19 (3.79)	29 (2.89)	1.13 (0.60–2.16)	0.689	0.81 (0.40–1.66)	0.570
Preschool	65 (12.97)	134 (13.37)	0.88 (0.49–1.59)	0.689	0.88 (0.48–1.61)	0.674
Citizenship**						
Not Senegalese	5 (1.0)	26 (2.59)	Ref.		Ref.	
Senegal	496 (99.00)	976 (97.41)	2.72 (1.02–7.26)	0.045	3.30 (1.20–9.09)	0.021
People sharing sleeping place**						
0–1	58 (11.58)	110 (10.98)	Ref.		Ref.	
2–3	400 (79.84)	853 (85.13)	0.89 (0.63–1.28)	0.555	0.98 (0.68–1.43)	0.927
> = 4	43 (8.58)	39 (3.89)	2.04 (1.15–3.62)	0.015	2.19 (1.18–4.05)	0.013
Household wealth index						
Low	268 (53.49)	508 (50.70)	Ref.		Ref.	
High	233 (46.51)	494 (49.30)	0.91 (0.68–1.21)	0.541	0.97 (0.71–1.32)	0.826
Main place of residence in study district**						
Yes	475 (94.81)	989 (98.70)	Ref.		Ref.	
No	26 (5.19)	13 (1.30)	6.10 (2.91–12.78)	0.000	3.27 (1.38–7.73)	0.007
Recruiting season**						
Low transmission	21 (4.19)	157 (15.67)	Ref.		Ref.	
High transmission	480 (95.81)	845 (84.33)	5.83 (3.52–9.63)	0.000	6.14 (3.67–10.26)	0.000
Recruiting district**						
Ranerou	36 (21.56)	70 (20.96)	Ref.		Ref.	
Kaolack	33 (19.76)	65 (19.46)	1.50 (1.13–2.00)	0.005	2.00 (1.31–3.06)	0.001
Saraya	98 (58.68)	199 (59.58)	0.96 (0.73–1.25)	0.772	1.15 (0.79–1.68)	0.474

^*^Adjusted for recruitment season and matching variables (age group, gender, and health facility); *Ref.* reference category

^**^Statistically significant results in multivariate logistic regression model

The district-specific models also showed similar results. The odds of being a nomadic pastoralist (OR 3.65 95% CI 1.36–9.82) (Supplementary Table 1) or gold miner (OR 2.40 95% CI 1.32–4.38) (Supplementary Table 3) was higher in cases than in controls in Ranérou and Saraya respectively. There was no evidence of an association with being a Koranic school student (OR 1.19 95% CI 0.59–2.41) in Kaolack (Supplementary Table 2).

The model combining the three districts and including all malaria risk factors considered to be on the causal pathway for the association between occupation and malaria (Supplementary Table 4), showed that having received IRS in the previous 12 months (OR 0.32 95%CI 0.15–0.71, compared to not having received it), having at least one LLIN for every 2 people (OR 0.59 95%CI 0.43–0.80) and having spent at least one night away from the current residence in the past month (OR 2.32 95%CI 1.22–4.40) were significant.

## Discussion

This study demonstrates that in Senegal, nomadic pastoralists and gold miners are at higher risk of malaria. They experience high exposure to mosquito bites both outdoors and indoors, and there is low coverage and use of LLIN, particularly among nomadic pastoralists. While other studies have established high malaria burden and identified related risk factors in these populations [14–16], this is the first study in this setting to confirm their specific occupation as an independent risk factor for malaria. Combined with the characterization of their exposure profile and the identification of gaps in coverage of malaria intervention, this study provides the Senegal NMCP with information needed to support policy formulations to target interventions appropriately to reduce malaria burden in these population subgroups.

While Koranic school learners were prioritized by the NMCP as a potential population at risk, and the formative assessment reported the existence of socio-behavioural risk factors for malaria in this group, this study did not find a significant association between symptomatic malaria infection and being a Koranic school learner. This could be explained by the fact that the case control study enrolment in Kaolack took place within a year of a universal LLIN mass distribution campaign that targeted all Koranic schools [19]. This is evidenced by a higher LLIN coverage and use among Koranic school learners compared to the non-HRP participants. Moreover, unlike the highly mobile nomadic pastoralists, Koranic school learners are a more fixed population and therefore more reachable by community-based interventions such as seasonal malaria chemoprevention when targeted appropriately.

In addition to identifying nomadic pastoralists and gold mining occupation as being at higher risk for malaria, this study also identified other established malaria risk factors. The risk of malaria was higher during the high transmission season, among participants sharing the sleeping space with four people or more in the household and having their main place of residence outside the study district [20–22]. Furthermore, this study identified factors mediating the association between the high-risk occupations and malaria infection. These mediators include not receiving IRS, not having enough LLINs and having travelled in the previous month and can be targeted to reduce malaria burden in these specific populations.

Malaria high risk populations have been identified in different settings including forest goers and mobile and migrant populations in Southeast Asia [23, 24]; gold miners in Latin America [10, 25, 26]; and male travellers to Angola in Namibia [7, 27], and different approaches have been piloted to address malaria transmission in these groups, especially in southeast Asia [28–30]. These populations subgroups share common behaviours, occupations and socio-demographics characteristics placing them at higher risk of malaria infection, due to increased exposure to infectious mosquito bites as a consequence of low vector control intervention coverage coupled with frequent outdoor exposure [12, 13, 31]. Some of these key characteristics were reported among the three populations during the formative assessment and confirmed by quantitative data from the case control study. Compared to the non-high risk participants, nomadic pastoralists and gold miners had a lower education level. Nomadic pastoralists were highly mobile lived in informal structures that were not sprayable and had a higher rate of outdoor sleeping. Koranic school students and nomadic pastoralists also spent more time outside between 6 pm and 6am than the non-high-risk groups participants. However, the low ownership and use of LLINs among these three population subgroups reported during the formative assessment was only confirmed by quantitative data for the nomadic pastoralists. For the Koranic school students and the gold miners these coverages were probably higher due to the LLINs distribution campaign organized the year of data collection. Similarly, the low access to healthcare services reported during the formative assessment was confirmed by the quantitative data only for the Koranic school students and gold miners.

The results of this study have important programmatic implications for the design and implementation of interventions targeting high risk populations. While the three population subgroups have common characteristics, they have different exposures, gaps, and barriers to intervention that will require targeted interventions to be tailored to their specific needs. Although the association between malaria and being Koranic school students was not statistically significant in this study, this population subgroup has clear risk factors and represents a challenge for the NMCP, therefore they should not be excluded from specific targeted programmes [27–29]. Based on the results of this study, challenges, limitations and opportunities for different targeted interventions have been identified. IRS, where applicable, wouldn’t be suitable for gold miners who sleep in unsprayable structures or for the highly mobile nomadic pastoralists. In contrast, IRS would provide protection to Koranic school students, as the schools are usually in sprayable buildings. LLINs would be more suitable for gold miners and Koranic school students, although the challenge of needing larger LLINs to cover the large sleeping spaces at the Koranic school would need to be addressed. The U.S. President’s Malaria Initiative (PMI) insights project and partners are conducting a study to evaluate the effect of an intervention package on malaria in Daaras in including the use of meganets [32]. Alternative vector control measures such as topical or spatial repellents, insecticide-treated clothing or LLINs with stronger fabrics would be needed to address the high rate of outdoor exposure among nomadic pastoralists, although making these interventions sustainable might be challenging. Based on the recent WHO recommendations [33], drug-based interventions, such as targeted drug administration (TDA) could be considered to reduce malaria burden among high-risk populations and eventually reduce malaria transmission. This strategy has been shown to be effective in reducing malaria prevalence and malaria incidence in similar populations [30, 34]. The information on their movement patterns collected during the formative assessment can inform operations planning on where and when to access these populations. Lastly, to address the low access to health services, an enhanced community case management programme, ideally using peer community health workers, would be needed. Such approach has been piloted by the Senegal NMCP through the PECADOM (“home-based case management”) Ferlo initiative, which deployed a community health worker in every village in Ranérou district to conduct passive case management and active weekly screening for fever. A pastoralist PECADOM component was also implemented, where one pastoralist from each group travelling together was trained as a CHW to be able to test and treat malaria cases.

A number of limitations associated with the study design and the implementation are noted. Both cases and controls were recruited at the health facilities, therefore there may be an association between health-seeking behaviours and the different population categories, especially among high-risk populations, that could confound the relationship with clinical malaria. Despite the fact that cases and controls' exposure status (high risk occupation) influences their likelihood of being recruited in the study via their health-seeking behaviour, both cases and controls are drawn from the same source population, reducing the risk of selection bias. Second, it is possible that information on specific exposures such as occupation may not have been accurate. For example, since a Koranic school is considered as a formal school to some extent, the difference between students and Koranic school learners may not have been captured, leading to possible exposure misclassification bias that may have an impact on estimating accurately the association between Koranic school learners and clinical malaria. Additionally, collecting self-reported information on household assets and malaria control interventions, especially in mobile populations, carries potential risk of recall bias. Finally, despite all the necessary adjustments made in the analysis, there’s still a risk of residual confounding. While high-risk populations may have similar underpinning characteristics to those found in other settings, the populations identified in this study are unique to the study settings, with specific behaviours, occupations, and sociodemographic characteristics, limiting the generalizability of these findings.

The main strength of this study included the mixed methods design. Collection of qualitative data prior to the case control study and immediate integration of results into study procedures allowed the refinement of data collection tools and provided a better understanding of the individual and social context to support the interpretation of the case control study results.

## Conclusions

In Senegal, nomadic pastoralists and gold miners are at higher risk of malaria. Compared to the non-HRP participants, they have a higher indoor and outdoor exposure to mosquito bites with limited access to health services. These findings suggest the need for targeted interventions to cover gaps in malaria prevention coverage and access to health services among these populations. Planning for tailored interventions should consider the specific behaviours and characteristics of each group identified in this study to ensure successful implementation. Future work should focus on evaluating the impact and feasibility of targeted interventions to reduce the malaria burden among these high-risk populations.

## Supplementary Information

Supplementary Material 1

## Data Availability

The data set supporting the results of this article will be made available upon request.
