# Supplementary material for: Identifying populations at high risk of malaria: a mixed-methods case–control study to inform targeted interventions in Senegal
Source: Malar J. 2024 Dec 18;23:373. doi: 10.1186/s12936-024-05219-z (PMC11657195; doi:10.1186/s12936-024-05219-z)
Supplement: Supplementary file 1 — Supplementary Material 1 [file 12936_2024_5219_MOESM1_ESM.docx]

Supplementary table 1: **Bivariate* and multivariable logistic regression model results** for the Ranérou Ferlo district

|  | Cases  n(%)  (N=167) | Controls n(%)  (N=334) | OR* (95% CI) | *p -* value | Adjusted OR (95% CI) | *p*- value |
| --- | --- | --- | --- | --- | --- | --- |
| Age group |  |  |  |  |  |  |
| 0-4 y | 18 (10.78) | 36 (10.78) | ref |  | ref |  |
| 5-14 y | 49 (29.34) | 98 (29.34) | 1.05 (0.54 - 2.05) | 0.875 | 0.40 (0.09 -1.77) | 0.227 |
| 15-24 y | 50 (29.94) | 112 (33.53) | 0.90 (0.46 - 1.74) | 0.747 | 0.29 (0.06 - 1.41) | 0.124 |
| 25-49 y | 42 (24.15) | 72 (21.56) | 1.17 (0.59 - 2.34) | 0.639 | 0.29 (0.06 - 1.47) | 0.136 |
| ≥50 y | 8 (4.79) | 16 (4.79) | 1.03 (0.37 - 2.89) | 0.942 | 0.37 (0.06 - 2.25) | 0.281 |
| Gender |  |  |  |  |  |  |
| Male | 100 (59.88) | 200 (59.88) | ref |  | ref |  |
| Female | 67 (40.12) | 134 (40.12) | 1.00 (0.68 - 1.46) | 0.01 | 1.08 (0.71 - 1.64) | 0.722 |
| Occupation |  |  |  |  |  |  |
| Non-nomadic pastoralist | 139 (82.23) | 324 (97.00) | ref |  | ref |  |
| Nomadic pastoralist | 28 (16.77) | 10 (3.00) | 8.22(3.67 - 18.40) | 0.000 | 3.65 (1.36 - 9.82) | 0.010 |
| Education level |  |  |  |  |  |  |
| No formal education | 121 (72.46) | 254 (76.05) | ref |  | ref |  |
| Primary school | 20 (11.98) | 24 (7.19) | 1.82 (0.88 - 3.78) | 0.10 | 1.37 (0.60 - 3.15) | 0.452 |
| Secondary school | 1 (0.60) | 4 (1.20) | 0.58 (0.06 - 5.48) | 0.64 | 0.56 (0.06 - 5.39) | 0.617 |
| Koranic school | 4 (2.40) | 3 (0.90) | 2.74 (0.59 - 12.64) | 0.19 | 0.70 (0.11 - 4.67) | 0.713 |
| Preschool | 21 (12.57) | 49 (14.67) | 0.50 (0.13 - 1.94) | 0.32 | 0.40 (0.09 - 1.67) | 0.206 |
| Family member diagnosed of malaria in past days |  |  |  |  |  |  |
| No | 129 (77.25) | 266 (79.64) | ref |  | ref |  |
| Yes | 33 (19.76) | 39 (11.68) | 1.75 (1.01 - 3.03) | 0.046 | 1.41 (0.76 - 2.60) | 0.275 |
| Don’t know | 5 (2.99) | 29 (8.68) | 0.30 (0.10 - 0.85) | 0.024 | 0.33 (0.11 - 0.96) | 0.042 |
| Malaria diagnosis in past month |  |  |  |  |  |  |
| No | 126 (75.45) | 284 (85.03) | ref |  | ref |  |
| Yes | 41 (24.55) | 50 (14.97) | 7.02 (2.54 - 19.35) | 0.000 | 5.94 (2.03 - 17.40) | 0.001 |
| Number of sleeping spaces in household |  |  |  |  |  |  |
| 0 - 3 places | 51 (30.54) | 74 (22.16) | ref |  | ref |  |
| >= 4 places | 116 (69.46) | 260 (77.84) | 0.62 (0.40 - 0.95) | 0.027 | 0.96 (0.47 - 1.92) | 0.897 |
| Household wealth index |  |  |  |  |  |  |
| Low | 162 (97.01) | 323 (96.71) | ref |  | ref |  |
| High | 5 (2.99) | 11 (3.29) | 0.88 (0.30 - 2.60) | 0.817 | 0.57 (0.16 - 2.06) | 0.387 |
| Main place of residence in study district |  |  |  |  |  |  |
| Yes | 143 (85.63) | 321(96.11) | ref |  | ref |  |
| No | 24 (14.37) | 13 (3.89) | 5.92 (2.63 - 13.29) | 0.000 | 3.68 (1.23 - 10.99) | 0.020 |
| Household size |  |  |  |  |  |  |
| 0-5 people | 28 (16.77) | 31 (9.28) | ref |  | ref |  |
| 6-7 people | 29 (17.37) | 47 (14.07) | 0.62 (0.30 - 1.25) | 0.178 | 0.87 (0.37 - 2.04) | 0.743 |
| 8-10 people | 40 (23.95) | 117 (35.03) | 0.35 (0.18 - 0.66) | 0.001 | 0.63 (0.25 - 1.60) | 0.333 |
| >=11 people | 70 (41.92) | 139 (41.62) | 0.51 (0.27 - 0.93) | 0.029 | 1.03 (0.39 -2.71) | 0.960 |
| Recruiting season |  |  |  |  |  |  |
| Low transmission | 2 (1.20) | 12 (3.59) | ref |  | ref |  |
| High transmission | 165 (98.80) | 322 (96.41) | 3.15 (0.68 - 14.47) | 0.139 | 13.32 (1.95 - 90.86) | 0.008 |
| Recruiting health facility |  |  |  |  |  |  |
| Mbem Mbem | 36 (21.56) | 70 (20.96) | ref |  | ref |  |
| Naouré | 33 (19.76) | 65 (19.46) | 0.95 (0.52 - 1.73) | 0.890 | 0.17 (0.05 - 0.54) | 0.003 |
| Salalatou | 98 (58.68) | 199 (59.58) | 0.96 (0.60 - 1.55) | 0.895 | 0.81 (0.45 - 1.47) | 0.491 |

*adjusted for recruitment season and matching variables (age group, gender, and health facility); ref: reference category

Supplementary table 2: **Bivariate* and multivariable logistic regression model results** for the Kaolack district

|  | Cases  n(%)  (N=167) | Controls n(%)  (N=334) | OR* (95% CI) | *p -* value | Adjusted  OR (95% CI) | *p*- value |
| --- | --- | --- | --- | --- | --- | --- |
| Age group |  |  |  |  |  |  |
| 0-4 y | 4 (2.40) | 8 (2.40) | ref |  | ref |  |
| 5-14 y | 63 (37.72) | 126 (37.72) | 1.03 (0.27 - 3.92) | 0.961 | 0.72 (0.14 - 3.61) | 0.688 |
| 15-24 y | 48 (28.74) | 99 (29.64) | 0.82 (0.21 - 3.17) | 0.784 | 0.69 (0.12 - 3.82) | 0.670 |
| 25-49 y | 38 (22.75) | 73 (21.86) | 0.74 (0.18 - 2.92) | 0.671 | 0.52 (0.09 - 2.89) | 0.457 |
| ≥50 y | 14 (8.38) | 28 (8.38) | 0.75 (0.17 - 3.27) | 0.708 | 0.50 (0.08 - 3.05) | 0.452 |
| Gender |  |  |  |  |  |  |
| Male | 106 (63.47) | 212 (63.47) | ref |  | ref |  |
| Female | 61 (36.53) | 122 (36.53) | 0.77 (0.50 - 1.19) | 0.251 | 0.90 (0.56 - 1.44) | 0.664 |
| Occupation |  |  |  |  |  |  |
| Non-Koranic school student | 121 (72.46) | 279 (83.53) | ref |  | ref |  |
| Koranic school student | 46 (27.54) | 55 (16.47) | 2.11 (1.19 - 3.74) | 0.010 | 1.19 (0.59 - 2.41) | 0.622 |
| Education level |  |  |  |  |  |  |
| No formal education | 73 (43.71) | 99 (29.53) | ref |  | ref |  |
| Primary school | 43 (25.75) | 112 (33.53) | 0.46 (0.26 - 0.80) | 0.007 | 0.59 (0.32 - 1.10) | 0.098 |
| Secondary school | 29 (17.37) | 81 (24.25) | 0.41 (0.22 - 0.76) | 0.005 | 0.49 (0.26 - 0.95) | 0.034 |
| University | 2 (1.20) | 9 (2.69) | 0.27 (0.05 - 1.45) | 0.129 | 0.29 (0.05 - 1.62) | 0.160 |
| Koranic school | 10 (5.99) | 12 (3.59) | 0.81 (0.28 - 2.30) | 0.693 | 0.22 (0.06 - 0.85) | 0.028 |
| Preschool | 10 (5.99) | 21 (6.29) | 0.55 (0.18 - 1.70) | 0.308 | 0.64 (0.20 - 2.08) | 0.459 |
| People sharing sleeping place |  |  |  |  |  |  |
| 0-1 | 17 (10.18) | 38 (11.38) | ref |  | ref |  |
| 2-3 | 126 (75.45) | 284 (85.03) | 1.02 (0.53 - 1.96) | 0.944 | 0.95 (0.48 - 1.89) | 0.888 |
| >=4 | 24 (14.37) | 12 (3.59) | 4.40 (1.63 - 11.87) | 0.003 | 3.38 (0.96 - 11.88) | 0.057 |
| Family member diagnosed of malaria in past days |  |  |  |  |  |  |
| No | 129 (77.25) | 285 (85.33) | ref |  | ref |  |
| Yes | 22 (13.17) | 27 (8.08) | 1.70 (0.87 - 3.31) | 0.119 | 1.22 (0.57 - 2.60) | 0.603 |
| Don’t know | 16 (9.58) | 22 (6.59) | 2.58 (1.14 - 5.80) | 0.022 | 2.84 (1.23 - 6.59) | 0.015 |
| Household wealth index |  |  |  |  |  |  |
| Low | 55 (32.93) | 46 (13.77) | ref |  | ref |  |
| High | 112(67.07) | 288 (86.23) | 0.38 (0.23 - 0.63) | 0.000 | 0.48 (0.27 - 0.85) | 0.013 |
| Number of household members |  |  |  |  |  |  |
| 0-5 | 35 (20.96) | 96 (28.74) | ref |  |  |  |
| 6-7 | 40 (23.95) | 93 (27.84) | 1.24 (0.69 -2.21) | 0.469 | 1.33 (0.72 - 2.44) | 0.358 |
| 8-10 | 37 (22.16) | 67 (20.06) | 1.28 (0.70 - 2.34) | 0.427 | 1.48 (0.77 - 2.82) | 0.238 |
| >=11 | 55 (32.93) | 78 (23.35) | 1.79 (1.00 - 3.18) | 0.049 | 1.64 (0.86 - 3.13) | 0.134 |
| Recruiting season |  |  |  |  |  |  |
| Low transmission | 19 (11.38) | 145 (43.41) | ref |  | ref |  |
| High transmission | 148 (88.62) | 189 (56.59) | 7.73 (4.41 - 13.57) | 0.000 | 7.30 (4.02 - 13.3) | 0.000 |
| Recruiting health facility |  |  |  |  |  |  |
| Ngane | 80 (23.95) | 40 (23.95) | ref |  | ref |  |
| Nimzatt | 100 (29.94) | 50 (29.94) | 0.58 (0.32 - 1.04) | 0.070 | 0.49 (0.24 - 0.99) | 0.045 |
| Thioffac | 94 (28.14) | 47 (28.14) | 0.54 (0.30 - 0.98) | 0.044 | 0.54 (0.28 - 1.02) | 0.058 |
| Taba Ngoye | 60 (17.96) | 30 (17.96) | 0.88 (0.46 - 1.67) | 0.699 | 0.90 (0.45 - 1.78) | 0.759 |

*adjusted for recruitment season and matching variables (age group, gender, and health facility); ref: reference category

Supplementary table 3: **Bivariate* and multivariable logistic regression model results** for the Saraya district

|  | Cases  n(%)  (N=167) | Controls n(%)  (N=334) | OR* (95% CI) | *p -* value | Adjusted OR  (95% CI) | *p*- value |
| --- | --- | --- | --- | --- | --- | --- |
| Age group |  |  |  |  |  |  |
| 0-4 y | 30 (17.96) | 60 (17.96) | ref |  | ref |  |
| 5-14 y | 66 (39.52) | 132 (39.52) ) | 1.00 (0.58 - 1.70) | 0.993 | 0.93 (0.39 - 2.21) | 0.864 |
| 15-24 y | 41 (24.55) | 71 (21.26) | 1.16 (0.64 - 2.09) | 0.622 | 1.02 (0.33 - 3.15) | 0.968 |
| 25-49 y | 26 (15.57) | 63 (18.86) | 0.82 (0.43 - 1.55) | 0.557 | 0.73 (0.23 - 2.32) | 0.592 |
| ≥50 y | 4 (2.40) | 8 (2.40) | 0.99 (0.27 - 3.60) | 0.993 | 0.88 (0.18 - 4.37) | 0.873 |
| Gender |  |  |  |  |  |  |
| Male | 89 (53.29) | 178 (53.29) | ref |  | ref |  |
| Female | 78 (46.71) | 156 (46.71) | 0.98 (0.67 - 1.44) | 0.928 | 1.23 (0.81 - 1.87) | 0.336 |
| Occupation |  |  |  |  |  |  |
| Non- gold miner | 114 (68.26) | 257 (76.95) | ref |  | ref |  |
| Gold miner | 53 (31.74) | 77 (23.05) | 2.82 (1.28 - 4.08) | 0.005 | 2.40 (1.32 - 4.38) | 0.004 |
| Education level |  |  |  |  |  |  |
| No formal education | 58 (34.73) | 124 (37.13) | ref |  | ref |  |
| Primary school | 62 (37.13) | 108 (32.34) | 1.45 (0.79 - 2.66) | 0.222 | 1.43 (0.76 - 2.66) | 0.265 |
| Secondary school | 8 (4.79) | 24 (7.19) | 0.73 (0.30 - 1.79) | 0.504 | 0.76 (0.30 - 1.89) | 0.550 |
| Koranic school | 5 (2.99) | 14 (4.19) | 0.93 (0.27 - 3.11) | 0.909 | 1.14 (0.33 - 3.92) | 0.840 |
| Preschool | 34 (20.36) | 64 (19.16) | 1.54 (0.57 - 4.17) | 0.392 | 1.37 (0.50 - 3.76) | 0.542 |
| Citizenship |  |  |  |  |  |  |
| Non-Senegalese | 5 (2.99) | 26 (7.78) | ref |  | ref |  |
| Senegalese | 162 (97.01) | 308 (92.22) | 2.78 (1.03 - 7.52) | 0.043 | 2.75 (0.98 - 7.70) | 0.055 |
| Household wealth index |  |  |  |  |  |  |
| Low | 51 (30.54) | 139 (41.62) | ref |  | ref |  |
| High | 116 (69.46) | 195 (58.38) | 1.64 (1.10 - 2.45) | 0.014 | 1.51 (1.00 - 2.29) | 0.052 |
| Recruiting health facility |  |  |  |  |  |  |
| Diakhaling | 83 (49.70) | 166 (49.70) | ref |  | ref |  |
| Khossanto | 84 (50.30) | 168 (50.30) | 0.99 (0.68 - 1.44) | 0.961 | 0.76 (0.50 - 1.15) | 0.198 |

*adjusted for recruitment season and matching variables (age group, gender, and health facility); ref: reference category

Supplementary table 4: **Bivariate* and multivariable logistic regression model results** for the three districts combined and including the malaria risk factor mediators (for the association between occupation and malaria)

|  | Cases n(%)  (N=501) | Controls n(%)  (N=1002) | OR* (95% CI) | *p -* value | Adjusted OR (95% CI) | *p*- value |
| --- | --- | --- | --- | --- | --- | --- |
| Age group |  |  |  |  |  |  |
| 0-4 y | 52 (10.38) | 104 (10.38) | ref |  | ref |  |
| 5-14 y | 178 (35.53) | 356 (35.53) | 1.07 (0.77- 1.57) | 0.725 | 0.91 (0.48 - 1.73) | 0.786 |
| 15-24 y | 139 (27.74) | 282 (28.14) | 0.99 (0.66 - 1.48) | 0.978 | 0.90 (0.43 - 1.88) | 0.780 |
| 25-49 y | 106 (21.16) | 208 (20.76) | 0.96 (0.63 - 1.46) | 0.874 | 0.79 (0.37 - 1.66) | 0.524 |
| ≥50 y | 26 (5.19) | 52 (5.19) | 0.91 (0.49 - 1.65) | 0.758 | 0.87 (0.36 - 2.07) | 0.744 |
| Gender |  |  |  |  |  |  |
| Male | 295 (58.88) | 590 (58.88) | ref |  | ref |  |
| Female | 206 (41.12) | 412 (41.12) | 0.92 (0.74 - 1.15) | 0.508 | 1.10 (0.87 - 1.41) | 0.431 |
| Occupation- Nomadic pastoralist |  |  |  |  |  |  |
| Non-Nomadic pastoralist | 473 (94.1) | 992 (99.0) | ref |  | ref |  |
| Nomadic pastoralist | 28 (5.9) | 10 (1.0) | 8.27 (3.60 - 18.99) | 0.000 | 1.52 (0.53- 4.37) | 0.432 |
| Occupation – Gold miners |  |  |  |  |  |  |
| Non-gold miner | 448 (89.42) | 925 (92.32) | ref |  | ref |  |
| Gold miner | 53 (10.58) | 77 (7.68) | 1.65 (0.97 - 2.81) | 0.061 | 2.21 (1.34 - 3.66) | 0.002 |
| Occupation – Koranic school student |  |  |  |  |  |  |
| Non-Koranic school student | 454 (90.62) | 947 (94.5) | ref |  | ref |  |
| Koranic school student | 47 (9.38) | 55 (5.49) | 1.54 (0.83 - 2.88) | 0.169 | 1.33 (0.76 - 2.35) | 0.318 |
| Education level |  |  |  |  |  |  |
| No formal education | 252 (50.30) | 477 (47.60) | ref |  | ref |  |
| Primary school | 125 (24.35) | 244 (24.35) | 0.97 (0.70 - 1.34) | 0.872 | 1.05 (0.74 - 1.50) | 0.778 |
| Secondary school | 38 (7.58) | 109 (10.88) | 0.62 (0.39 - 0.96) | 0.036 | 0.73 (0.45 - 1.18) | 0.194 |
| University | 2 (0.40) | 9 (0.90) | 0 .41 (0.08 - 2.05) | 0.280 | 0.35 (0.07 - 1.85) | 0.216 |
| Koranic school | 19 (3.79) | 29 (2.89) | 1.13 (0.60 - 2.16) | 0.689 | 0.68 (0.32 - 1.44) | 0.315 |
| Preschool | 65 (12.97) | 134 (13.37) | 0.88 (0.49 - 1.59) | 0.689 | 0.82 (0.44 - 1.55) | 0.551 |
| Citizenship |  |  |  |  |  |  |
| Non Senegalese | 5(1.0) | 26(2.59) | ref |  | ref |  |
| Senegalese | 496 (99.00) | 976 (97.41) | 2.72 (1.02 - 7.26) | 0.045 | 3.24 (1.16 - 9.06) | 0.025 |
| People sharing sleeping space |  |  |  |  |  |  |
| 0-1 | 58 (11.58) | 110 (10.98) | ref |  | ref |  |
| 2-3 | 400 (79.84) | 853 (85.13) | 0.89 (0.63 - 1.28) | 0.555 | 0.95 (0.64 - 1.40) | 0.779 |
| >=4 | 43 (8.58) | 39 (3.89) | 2.03 (1.14 - 3.61) | 0.015 | 1.81 (0.95 - 3.42) | 0.069 |
| Household wealth index |  |  |  |  |  |  |
| Low | 268 (53.49) | 508 (50.70) | ref |  | ref |  |
| High | 233 (46.51) | 494 (49.30) | 0.91 (0.68 - 1.21) | 0.541 | 1.02 (0.74 - 1.40) | 0.909 |
| Main place of residence in the study district |  |  |  |  |  |  |
| No | 475 (94.81) | 989 (98.70) | ref |  | ref |  |
| Yes | 26 (5.19) | 13 (1.30) | 6.10 (2.91 - 12.78) | 0.000 | 1.99 (0.79 - 5.06) | 0.147 |
| Indoor residual spraying (IRS) |  |  |  |  |  |  |
| No | 42 (8.38) | 19 (1.90) | ref |  | ref |  |
| Yes | 150 (29.94) | 367 (36.63) | 0.15 (0.08 - 0.28) | 0.000 | 0.32 (0.15 - 0.71) | 0.005 |
| Not applicable | 309 (61.68) | 616 (61.48) | 0.16 (0.07 - 0.36) | 0.000 | 0.27 (0.10 - 0.70) | 0.008 |
| Seasonal malaria chemoprevention (SMC) |  |  |  |  |  |  |
| No | 70 (13.97) | 96 (9.58) | ref |  | ref |  |
| Yes | 52 (10.38) | 117 (11.68) | 0.65 (0.41 - 1.05) | 0.080 | 0.69 (0.42 - 1.13) | 0.143 |
| Not applicable | 379 (75.65) | 789 (78.74) | 0.54 (0.35 - 0.84) | 0.006 | 0.60 (0.38 - 0.96) | 0.034 |
| Ratio LLIN per person |  |  |  |  |  |  |
| Less than 1 LLIN for 2 people | 381 (76.05) | 659 (65.77) | ref |  | ref |  |
| At least 1 LLIN for 2 people | 120 (23.95) | 343 (34.23) | 0.60 (0.46 - 0.78) | 0.000 | 0.59 (0.43 - 0.80) | 0.001 |
| Mosquito net ownership |  |  |  |  |  |  |
| No | 165 (32.93) | 270 (26.95) | ref |  | ref |  |
| Yes | 336 (67.07) | 732 (73.05) | 0.72 (0.55 - 0.93) | 0.014 | 0.75 (0.50 - 1.13) | 0.168 |
| Slept under LLIN last night |  |  |  |  |  |  |
| No | 233 (46.51) | 421 (42.02) | ref |  | ref |  |
| Yes | 268 (53.49) | 581 (57.89) | 0.83 (0.65 - 1.05) | 0.128 | 1.25 (0.87 - 1.79) | 0.230 |
| Traveled in the last month |  |  |  |  |  |  |
| No | 466 (93.01) | 981 (97.90) | ref |  | ref |  |
| Yes | 35 (6.99) | 21 (2.10) | 3.42 (1.93 - 6.04) | <0.001 | 2.32 (1.22 - 4.40) | 0.010 |
| Slept outside in the last month |  |  |  |  |  |  |
| No | 381 (76.05) | 818 (81.64) | ref |  | ref |  |
| Yes | 120 (23.95) | 184 (18.36) | 1.44 (1.09 - 1.91) | 0.010 | 1.31 (0.92 - 1.86) | 0.130 |
| Weekly hours spent outside between 18h and 6h in the past month |  |  |  |  |  |  |
| No time spent outside | 88 (17.56) | 191 (19.06) | ref |  | ref |  |
| 1-12 h | 224 (44.71) | 491 (49.00) | 0.97 (0.69 - 1.38) | 0.901 | 0.77 (0.49 - 1.19) | 0.241 |
| 13-16 h | 88 (17.56) | 188 (18.76) | 1.02 (0.67 - 1.56) | 0.899 | 0.74 (0.45 - 1.22) | 0.236 |
| ≥16 h | 101 (20.16) | 132 (13.17) | 1.75 (1.14 - 2.67) | 0.009 | 1.08 (0.64 - 1.80) | 0.776 |
| Recruiting season |  |  |  |  |  |  |
| Low transmission | 21 (4.19) | 157 (15.67) | ref |  | ref |  |
| High transmission | 480 (95.81) | 845 (84.33) | 5.83 (3.52 - 9.63) | <0.001 | 5.68 (3.37 - 9.56) | 0.000 |
| Recruiting district |  |  |  |  |  |  |
| Ranerou | 36 (21.56) | 70 (20.96) | ref |  | ref |  |
| Kaolack | 33 (19.76) | 65 (19.46) | 1.50 (1.13 - 2.00) | 0.005 | 2.94 (1.35 - 6.37) | 0.006 |
| Saraya | 98 (58.68) | 199 (59.58) | 0.96 (0.73 - 1.25) | 0.772 | 1.77 (0.90 - 3.51) | 0.100 |

*adjusted for recruitment season and matching variables (age group, gender, and health facility); ref: reference category
